# Opioid Impacts on Cardiovascular Health

**DOI:** 10.7759/cureus.46224

**Published:** 2023-09-29

**Authors:** Erjola Toska, Harvey N Mayrovitz

**Affiliations:** 1 Osteopathic Medicine, Nova Southeastern University Dr. Kiran C. Patel College of Osteopathic Medicine, Fort Lauderdale, USA; 2 Medical Education, Nova Southeastern University Dr. Kiran C. Patel College of Allopathic Medicine, Fort Lauderdale, USA

**Keywords:** naloxone, opioid addiction, coronary heart disease, hypertension, opioid overdose, opioid withdrawal, opioid use, stroke, arterial stiffness, vascular aging

## Abstract

The prevalence of opioid use in the current opioid epidemic era has led to a public health emergency due to the ties to mortality and morbidity. Studies have investigated opioids’ impacts on different aspects of cardiovascular health, although there seems to be a lack of a current concise review. Therefore, the aim of this literature review is to provide a summary of the most recent studies from the past decade that postulate a connection between opioids and their impact on cardiovascular health while highlighting conflicting areas among published research. For this literature review, three databases, PubMed (NLM), EMBASE, and Web of Science (Core Collection), were searched for full peer-reviewed articles written in English about human subjects and published between 2013 and 2023 inclusive. The following initial approach was to search for terms in the title of articles: “opioid AND (“vascular” OR “artery” OR “vein” OR “heart rate” OR “infarct” OR “stroke” OR “aortic” OR “cardiovascular disease”). After assessing for duplicate articles from the three databases, the remaining articles were assessed for inclusion eligibility. In the present review, a brief description of the overall role of opioid receptors is provided followed by the literature findings. These findings indicate potentially important negative impacts of opioid use on cardiovascular health in a number of areas. These include opioid-associated increases in the following: (1) vascular aging based on demonstrated increases in arterial stiffness, (2) opioid-related reductions in heart rate variability (HRV) and its implications on morbidity and mortality, (3) opioid’s impacts on coronary artery and coronary heart disease (CHD), (4) opioids as a risk factor for atrial fibrillation (AF) and (5) opioid use as a risk factor for vascular occlusion processes. In addition to these broad cardiovascular effects, other aspects of concern are related to the potential impacts of withdrawal from opioid use, which, when done rapidly, are associated with increases in blood pressure and a decrease in HRV.

## Introduction and background

Opioids are a class of drugs that are commonly used for their analgesic effects to modulate pain via their actions on the opioid system which is composed of opioid peptides and receptors [1]. Opioid receptors are widely dispersed throughout the bodily organs including the liver, heart, GI tract, and the central and peripheral nervous system [2]. The presence of receptors allows opioids to have several physiological and pathological effects on different bodily systems such as the nervous, respiratory, GI, and cardiovascular systems, the last of which is the focus and scope of this review [3]. For example, opioid receptors are spread throughout the nervous system which govern brain physiology at different points in neural processing, including autonomic, emotional, sensory, and cognitive functions, ultimately leading to analgesic effects [4]. In the respiratory system, opioids induce respiratory depression perceived by the regularity of the inspiratory rhythm and decreased frequency of inspiration [5]. The activation of opioid receptors in the GI tract inhibits the neuronal excitability of the enteric system decelerating the transit time of contents from the stomach to the small intestine while also exerting a suppressive influence on secretion and absorption of the GI tract [6].

Opioid use was stable for the two decades prior to 2008 at which time national trends saw a sharp increase in the United States (US) [7]. In 2018, it was reported that over 10 million people were misusing prescription opioids such as oxycodone, fentanyl, codeine, meperidine, morphine sulfate, and others [8-10]. The US government declared the growing opioid epidemic a public health emergency due to increased opioid-related deaths quintupling in the past 20 years and opioids linked to serious health issues [7,11]. In 2021, the World Health Organization disclosed that over 70% of fatalities linked to drug usage were attributed to opioids, with 30% of these fatalities arising from the physiological effects of opioids on the brain's respiratory regulation causing overdose deaths [12]. While researchers are still actively researching the different aspects of opioids, studies have shown some associations between opioids and cardiovascular health [8,13,14].

Cardiovascular disease (CVD) is a major contributor to global mortality, with an increasing prevalence from 271 million cases in 1990 to 523 million cases across 204 countries by 2019 [3,15,16]. The opioid epidemic has spurred research into the impacts of opioid use on cardiovascular health with the indication that its use may increase the risk of death from cardiovascular events [3,8]. Different studies have examined other factors about the potential negative impacts of opioid use on vascular features and function [17,18]. There is a present need to examine and integrate the overall opioid-cardiovascular connection and consolidate the current status of understanding both concerning the use of opioids and withdrawal from their use. The purpose of this review is to meet this need.

## Review

Methods

Three databases, PubMed (NLM), EMBASE, and Web of Science (Core Collection), were searched for full peer-reviewed articles written in English about human subjects and published between 2013 and 2023 inclusive. The following initial approach was to search for terms in the title of articles: “opioid AND (“vascular” OR “artery” OR “vein” OR “heart rate” OR “infarct” OR “stroke” OR “aortic” OR “cardiovascular disease”). After assessing for duplicate articles from the three databases, the remaining articles were assessed for inclusion eligibility. Studies were excluded if they were written in non-English languages, were animal studies, had only abstracts/poster presentations, were review articles, were commentaries, were editorials, and had a non-opioids/non-cardiovascular focus (i.e., opioids prescription medication limitation policies following postoperative vascular surgery, the sole use of non-opioids in surgical settings, geographic relations to opioids in social determinants of health, fetal/spinal cord studies). Therefore, the remaining articles were evaluated for this literature review.

Role of Delta and Kappa opioid receptors

Opioid receptors are a part of the G protein-coupled receptors (GPCRs) family which are responsible for triggering different signaling pathways including stimulation of mitogen-activated protein (MAP) kinases, regulating adenylyl cyclase activities and modulation of ion conductance [11,19]. The classical opioid receptors, μ- (MOR), δ- (DOR), or κ- (KOR), are distributed in the nervous system and, upon stimulation, have the effects of causing bradycardia, analgesia, and constipation [1,19]. The specific effects of each receptor’s activation in the cardiovascular system across different articles concluded conflicting outcomes as to whether opioid receptor activation exerts apoptotic or anti-apoptotic effects on cells such as human umbilical vein endothelial cells (HUVECs) and breast cancer cells [11,19,20].

Concerning cardiovascular effects, one aspect of opioid’s impact in long-term use is due to opioid receptor activation promoting increasing inflammatory responses and reactive oxygen species (ROS) that can lead to cardiovascular complications such as microvascular dysfunction and atherosclerosis [20]. This negative impact is to be differentiated from acute opioid receptor activation. This aspect has been investigated using HUVECs that were acutely exposed to activated KORs. This caused intracellular ROS levels to decrease indicating a reduction in oxidative stress.

In another study, the role of acute activation of DOR was investigated with a DOR agonist D-Ala2, D-Leu5-Enkephalin (DADLE) in human breast cancer cells that shared similar anti-apoptotic effects [19]. In that study, the acute activation of DOR-activated PI3K/Akt promoted cell survival. The proposed ability of DADLE to preserve cells through these anti-apoptotic ischemic (hypoxic) conditions may explain its effects on ischemic strokes by reducing cell death following ischemic events [1]. The significance of the DOR protective nature, by their capacity to maintain ionic balance under hypoxic/ischemic conditions, may also be tied to their cardiovascular system impacts, particularly relevant in the context of ischemic strokes [1].

The acute cellular protective nature associated with KOR or DOR stimulation can be reduced or eliminated in the presence of chronic opioid use. Chronic opioid use increases tolerance and decreases opioid receptor signaling causing receptor desensitization. This process causes a reduction or loss of the protective mechanism of reduced oxidative stress, thereby creating increased susceptibility to inflammatory damages and ultimately CVD [11].

Opioid impacts on arterial stiffness and hypertension

Arterial stiffness is a measure of the ability of arteries to expand in response to an increase in transmural pressure [21]. With aging and other conditions such as hypertension, arterial stiffness tends to increase [22]. A consequence of this is that the propagation velocity of pressure pulse waves in large arteries increases [23]. One result of this increased speed is that reflected pressure waves arrive back at the heart earlier than normal causing an increase in central arterial systolic blood pressure [23]. This augmented pressure causes an increase in ventricular afterload and is detrimental to cardiac function [24]. Recently developed methods allow for the measurement of effective arterial stiffness by measuring the speed with which the pulse pressure wave moves, and the greater the velocity, the greater the stiffness [23]. As such, these arterial stiffness measures, determined by pulse wave analysis (PWA), are sometimes used to assess the relative “physiological age” of the arterial system. Increased stiffness has a negative effect on cardiovascular health [25].

One study evaluated arterial stiffness and central arterial blood pressure using PWA in 1,263 patients divided into four treatment groups: methadone (opiate agonist, n=71), buprenorphine (partial opiate agonist, n=593), naltrexone (opiate antagonist, n=23), and a non-treated control (n=576) [18]. The study’s data indicated a pattern of increasing arterial stiffness and increasing vascular age as the level of lifetime opioid agonism increased among groups. For example, comparing subjects at the age of 60 years, the predicted vascular age, based on arterial stiffness values, was 67.40 (control group), 72.03 (partial opiate agonist), and 82.79 (opiate agonist) years, respectively, in the order of increasing opioid agonism depicting increasing vascular age with opioid agonism [18].

An extended analysis of the 1,263 patients divided by age considered 576 patients as clinical controls and 687 as opioid-dependent patients [17]. Their results indicated that arterial stiffness and, thus, vascular age were worse in opioid-dependent patients compared with opioid clinical controls. The mean calculated vascular ages were elevated by 1.97% in men and 13.43% in women [17].

Opioid impacts on angiogenesis

Angiogenesis is the process by which new blood vessels form and malignant tumors require angiogenesis to support the growth of the tumor [26,27]. Thus, substances or processes that promote angiogenesis associated with tumors would indicate a negative feature. It has been suggested that opioid use may impact the angiogenic process and that opioids promote tumor angiogenesis [19]. In that study, DOR was stimulated with a DOR agonist DADLE in human breast cancer cells (MCF-7) through cell treatment incubation. The stimulation process led to the activation of hypoxia-inducible factor 1α (HIF-1α). The activation of HIF-1a triggered the synthesis and release of diverse angiogenic factors and also the expression of cyclooxygenase-2 via phosphoinositide 3-kinase(PI3K)/Akt stimulation [19].

Opioid association with retinal vein occlusion

Retinal vascular occlusion has been linked to different cardiovascular factors. In the case of retinal blood flow reduction, occlusion may be due to retinal vein occlusion in a branch or central vein [28]. In a retrospective study of 380 cases of branch occlusion and 311 cases of central vein occlusion, it was found that for these 691 cases compared to 1,520 controls, there was an increased risk of vein occlusion in those who reported they had used opioids [29]. Reported mean odds ratios (OR) ranged from 1.98 for branch occlusions to 2.32 for central vein occlusions for those who used opioids.

Opioids and atrial fibrillation

Atrial fibrillation (AF) is one of the most common cardiac arrhythmias [30-32] and is a significant risk factor for stroke [33-35]. An investigation of a possible link between opioid use and AF was done on 30,239 participants [14]. In this study, medical information including blood tests and electrocardiograms was obtained, and opioid use was established during in-home visits with pill-bottle review by trained staff [14]. Analysis of the data indicated a statistically significant effect of opioid use on AF after accounting for confounding variables with an OR of 1.29 (95%CI, 1.11-1.51). A possible explanation of this association was offered by the authors. They speculated that it was related to endogenous opioid effects that open mitochondrial potassium adenosine triphosphate channels. This process would make the atrial myocytes susceptible to oxidative stress during episodes of ischemia, therefore, leading to the increased chance of AF.

Opioids and heart rate variability

Heart rate variability (HRV) is an indicator of cardiac neural control and is measured by determining the temporal variation in the times between adjacent heartbeats that can be expressed in terms of time variations or in terms of spectral frequency distributions [36]. In the frequency representation, the spectral distribution is standardly divided into frequency bands, commonly referred to as very low frequency (VLF), low frequency (LF), and high frequency (HF). The ranges of the VLF, LF, and HF bands are 0.003-0.04 Hz, 0.04-0.15 Hz, and 0.15 -0.4 Hz. The power of the HF band is thought to depend on the modulation of cardiac parasympathetic efferent nerve traffic [37], whereas the LF band is thought to reflect a composite of both sympathetic and parasympathetic activity but with the sympathetic component more dominant [38,39]. Consequently, a higher HF power is thought to indicate greater parasympathetic influence. This can be assessed as the ratio of HF/LF power or by the percentage of the total HRV spectral power due to HF. Based on this concept, the impact of opioid long-term use on HRV was evaluated in a group of 491 opioid users [40]. In that study, a comparison was made between those who were diagnosed with opioid use disorder (OUD, n=258) and those long-term opioid users without such a diagnosis (n=232). The relevant finding to the present study was that the OUD group had a significantly reduced HF-HRV percentage (48.0 ± 22.5 vs. 57.3 ± 22.8, p<0.001) [41]. Furthermore, regression analysis indicated an increasing difference between groups with increasing OUD severity.

Opioid withdrawal

Several studies have used changes in HRV to assess other aspects of opioid use and withdrawal [40,42-44]. Because HRV is linked to sympathetic and parasympathetic activity of the autonomic nervous system and opioid use affects HRV, it is worthwhile to consider to potential impact of withdrawal of opioid use. In a study of 10 male physiologically dependent opioid users in whom opioid withdrawal was induced by the administration of the opioid antagonist naloxone, several cardiovascular changes were observed [40]. In this study withdrawal signs were noted in all subjects 21 minutes post-naloxone administration. Associated cardiovascular changes, as measured from baseline, included significant increases in HR, systolic blood pressure, and diastolic blood pressure and decreases in HF-HRV.

The simulation of opiate withdrawal is also experienced by using short-acting opiates such as morphine, oxycodone, and heroin which require users to re-administer to avoid the aversive effects of opiate withdrawal [18]. The adverse effects of opiate withdrawal include hypertensive and other cardiovascular changes [18]. The clinical syndrome experienced from opiate withdrawal is characterized as a hyper-adrenergic state with tachycardia and reduced subendocardial blood perfusion with an associated increased cardiovascular age and arterial stiffness [17].

Opioids and coronary heart disease

A prospective study of over 29,000 participants evaluated the association between opioid prescription use and the occurrence of coronary heart disease (CHD), stroke, and cardiovascular-related death. Participants, who were followed for a median time of 5.2 years, had an increased adjusted hazard risk of CHD (1.38) and cardiovascular deaths (1.66) only for females. Although several limitations to this study have been described [9], the overall findings are important for consideration. The presence of CHD is often associated with CAD which itself is linked to the presence of coronary artery atherosclerosis [45,46]. It may be that there is a linkage between opioid use and increased vascular inflammation due to elevated levels of interleukin-1 in opioid users thereby predisposing them to CAD [3]. Two studies [3,18] that evaluated coronary risk factors found that individuals who had taken opioids or opioid-dependent patients had an elevated risk for CAD with a 2.2-fold increase (95 % CI 1.8-2.7) [18].

In a study that investigated the association between prescription opioid use (POU) and CHD and CVD, it was found that POU in females increased the risk of both CHD and CVD death [9]. For both genders, POU was associated with an increased risk with an HR of 1.24 (95% CI: 1.08-1.42) with a greater impact among women with HR=1.35 (95%CI: 1.12-1.64) [13]. These findings were consistent with another study in which women with POU were at higher risk for CVD death, with an HR of 1.43 (95% CI 1.12-1.84). Taken together these data suggest a gender-dependent cardiovascular impact of opioid use, although the explanation is unknown [8].

Delving into the underlying causes of CAD and CVD, a patient’s post-mortem reports analysis study focused on mortality’s relation to opioid use [47]. This study had a sample of 436 individuals who had died due to CVD pathologies. Vascular pathologies examined included the presence and extent of atherosclerosis, atheroma, fibrosis, hypertrophy, inflammation, and stenosis. There was an association between the presence of opioids in the system of the post-mortem reports and the total CVD severity level consistent with a significant relationship between opioids and CVD pathologies with inflammation and atheroma leading to atherosclerosis [47].

Opioids in intra-operative surgical setting

Opioids used in general anesthesia may negatively impact cardiovascular system hemodynamics. A study aimed at evaluating this effect compared general anesthesia with opioids vs. general anesthesia with perioperative thoracic epidural analgesia. This was done in patients who had off-pump coronary artery bypass [48]. The group that received the opioids experienced a significant increase in heart rate and mean arterial pressure as compared to the other group. The authors speculated that the increased stress state that the opioid group experienced opens the possibility of opioid-related myocardial infarction, arrhythmia, and pulmonary edema during coronary artery bypass graft surgery. In a different surgical setting (transcatheter aortic valve implantation), the relative impacts of using dexmedetomidine (DEX) vs. propofol-opioid (PO) sedation was evaluated with 157 patients in the DEX group and 140 patients in the PO group [49]. It was found that administration of PO sedation had higher levels of hypercapnia requiring pharmacologic hemodynamic and vasopressor support. These two reports indicate opioid use in intraoperative settings should be carefully considered. The potential mortality impact of long-term opioid use prior to undergoing vascular surgery was evaluated in a convenience sample of 164 patients [50]. In this study, only six patients reported opioid prior use but compared to the non-users, statistical analysis indicated a higher post-surgical mortality risk with an adjusted HR of 4.31 (95% CI: 1.77-10.55, p=0.001).

## Conclusions

The findings of this review indicate potentially important negative impacts of opioid use on cardiovascular health in several areas. These include opioid-associated increases in (1) vascular aging as assessed by increases in arterial stiffness, (2) opioid-related reductions in HRV and its implications for future morbidity and mortality, (3) opioid’s potential impacts on coronary artery and coronary heart disease (CHD), (4) as a risk factor for atrial fibrillation, and (5) as a risk factor for vascular occlusion processes. In addition to these broad cardiovascular effects, other aspects of concern are related to the potential impacts of withdrawal from opioid use, which, when done rapidly, are associated with increased blood pressure and a decrease in HRV.
